# Private Fees in Public Hospitals

**Published:** 1898-05-07

**Authors:** 


### PRIVATE FEES IN PUBLIC HOSPITALS.

A case which was recently tried in Dublin well illustrates the difficulties which arise from hospital rules being framed without recognising the existence of the paying patient or making any arrangement for his reception. A gentleman who was apparently quite able to pay for whatever help he received met with an accident in the street, and was taken to the Jervis Street Hospital, suffering from compound fracture of the leg. There he was attended by Dr. Cranny, one of the surgical staff of the hospital, who afterwards sought to recover £25 for fees which he considered were due to him for his attendance on the patient while in hospital. Mr. Lee, the patient, had paid to the hospital three guineas a week, which he considered had discharged all claims against him. From the evidence of the secretary it was clear that no fees were prescribed either in the public or the private wards, and in the end the jury found that the plaintiff did not render the services on the terms that the defendant should pay specially therefor, and judgment was accordingly entered for the defendant. This was clearly the only possible verdict. But it is quite clear that if the Jervis Street Hospital had possessed a paying annexe, or even if the principle of payment according to means had been definitely recognised in the rules, this sort of thing would never have happened. How great and serious is the difficulty which arises from the admission of well-to-do patients to hospitals may be judged of by the mean-spirited actions to which it appears sometimes to lead. Here we have a well-to-do man receiving board, lodging in a private ward, nursing presumably by special nurses, and attendance not only by a well-known member of the staff but by the resident surgeon of the hospital, and all for the ridiculous sum of three guineas a week—something like what would be charged at a second or third-class hydropathic institution without either nursing or medical attendance, and, in fact, without any meals served in his rooms. Clearly the patient did well out of the charity.

Why, then, did not the hospital charge more? Probably because it was a charity! Because no arrangements were made in its rules for any except charity patients, and hence, because it knew that it had no right to charge at all; so it just charged what it thought the patient would pay without grumbling, and threw the doctor over altogether.

One of our contemporaries, the *British Medical Journal*, says, in regard to this case: "This case . . . . shows well the danger and the unfairness of mixing up paid practice with our present hospital system." We, however, would answer that it is impossible to help such "mixing up" unless we are prepared to hand over for the treatment of the rich money which has been collected for the treatment of the poor, or refuse the rich altogether, which is impossible. What is to be done when a rich man meets with an accident and is carried off to the hospital? He cannot be refused admission. If, then, he is to be prevented from taking the money subscribed for the poor arrangements must be made for him to pay for what he receives, and if he pays the hospital he should pay the doctor also. The *British Medical Journal* solves the problem in this way: "In gratuitous hospitals a wealthy person accidentally admitted should be left to his own conscience as to the remuneration he may think himself bound to give, whether to the institution or to its honorary officers." We do not, however, like this system of tipping; we do not think that it is consistent with the honour or dignity of the medical profession that honorary officers should attend rich hospital patients in the expectation of a gratuity. Another of our contemporaries says: "As a matter of fact, it must be confessed, and ought to be recognised, that hospital physicians and surgeons do commonly accept fees from hospital patients who are able to pay, receiving such fees either before the patient is admitted or after he comes out, but such method of remuneration is always open to the objection that it is more or less surreptitious." We do not for a moment believe that such a system exists, or that hospital surgeons at all "commonly accept fees," and we think that the *Medical Press and Circular* shows a serious misapprehension of the facts of the case in making such a charge against leaders in the profession. If, however, such underhand irregularities do exist in Ireland as are described in the *Medical Press and Circular*, all the more important is it that the well-to-do patient should be recognised in hospital rules, and that his position should be properly defined. The judge who tried this case said that "there should be a set of rules in Dublin hospitals such as existed at St. Thomas's Hospital, London, where a man paid so much and received everything he required." But even in the absence of a separate building for paying patients there ought, as we have urged again and again, to be a rule that patients who can pay should pay according to their means, and that so soon as that payment exceeds the sum spent in their maintenance a certain considerable proportion of it should be set apart for the medical staff. An open, business-like arrangement such as this would do away with all necessity for the "tip" suggested by the *British Medical Journal*, and for the "surreptitious" methods of remuneration which the *Medical Press and Circular* maintains are now in vogue.

